# Three Decades of Amyloid Beta Synthesis: Challenges and Advances

**DOI:** 10.3389/fchem.2019.00472

**Published:** 2019-07-02

**Authors:** Johanes K. Kasim, Iman Kavianinia, Paul W. R. Harris, Margaret A. Brimble

**Affiliations:** ^1^School of Biological Sciences, The University of Auckland, Auckland, New Zealand; ^2^Maurice Wilkins Centre for Molecular Biodiscovery, The University of Auckland, Auckland, New Zealand; ^3^School of Chemical Sciences, The University of Auckland, Auckland, New Zealand

**Keywords:** Alzheimer's disease, dementia, neurodegenerative disorders, difficult peptide sequences, amyloid beta, peptide chemistry, solid phase peptide synthesis

## Abstract

Aggregation of the pathological amyloid beta (Aβ) isoform Aβ_1−42_ into senile plaques is a neuropathological hallmark of Alzheimer's disease (AD). The biochemical significance of this phenomenon therefore necessitates the need for ready access to Aβ_1−42_ for research purposes. Chemical synthesis of the peptide, however, is technically difficult to perform given its propensity to aggregate both on resin during solid phase peptide synthesis and in solution during characterization. This review presents a chronological summary of key publications in the field of Aβ_1−42_ synthesis, dating back from its maiden synthesis by Burdick et al. Challenges associated with the preparation of Aβ_1−42_ were identified, and the solutions designed over the course of time critically discussed herein. Ultimately, the intention of this review is to provide readers with an insight into the progress that has been made in the last three decades, and how this has advanced broader research in AD.

## Introduction

Alzheimer's disease (AD) is a degenerative disorder of the brain that was named after the German psychiatrist Alois Alzheimer, who in 1906 delivered a lecture at the 37th Conference of South-West German Psychiatrists in Tubingen, detailing his observations on a novel form of dementia that had befallen a 51-year-old Frankfurt woman by the name of Auguste Deter (Maurer et al., 1997). According to the latest World Health Organization (WHO) report, AD contributes to over two-thirds of dementia cases worldwide, as according to the latest World Health Organization (WHO) report (World Health Organization, 2019). On a histopathological level, the disease is characterized by two hallmarks: senile plaques and tangles (Scheltens et al., 2016). The former is composed of extraneuronal deposits of amyloid beta (Aβ) peptide, while the latter arises from intracellular interactions between hyperphosphorylated tau, a microtubule-associated protein that would normally facilitate cytoskeletal transport within the axonal network. Clinically, AD patients present with a progressive loss of cognitive function; this is because the hippocampus, which is the brain region associated with learning and memory processes, is one of the first affected structures in the course of disease progression (Halliday, 2017).

Research endeavors in AD over the last few decades have predominantly focussed on Aβ peptide, which was proposed by Hardy and Higgins in their “amyloid cascade hypothesis” to be a central figure in the overall disease mechanism (Hardy and Higgins, 1992). While this hypothesis has come under substantial criticism recently (Herrup, 2015), in light of a number of high-profile failures in clinical trials for novel AD drugs (Cummings et al., 2018), the role of Aβ peptide in the disease, regardless of its actual extent, remains pivotal toward understanding the complex underlying architecture of AD.

**Figure 1:**
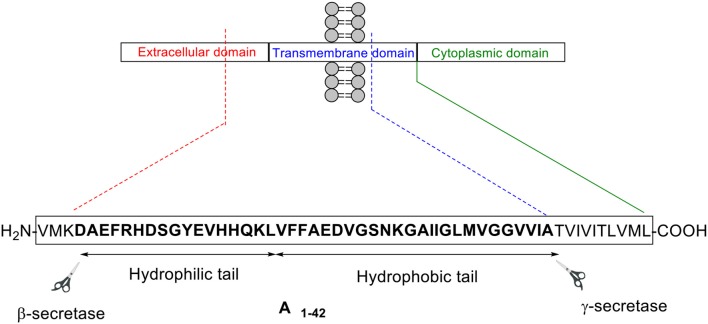
Aβ peptide is generated from APP processing via the amyloidogenic pathway.

Aβ peptide was first isolated in 1984 from a larger precursor molecule named amyloid precursor protein (APP) (Glenner and Wong, 1984). It is understood that APP is processed by the secretase family of enzymes via two main pathways: non-amyloidogenic and amyloidogenic (Figure 1; Thinakaran and Koo, 2008)

In the former pathway, α-secretase cleaves APP between residues Lys16 and Leu17 in the Aβ encoding region. This cleavage, which generates two fragments—soluble APP alpha (sAPPα) and C83, an 83-residue long C-terminus fragment, effectively disables formation of intact Aβ peptide, hence the designated term “non-amyloidogenic.” γ-secretase then subsequently cleaves C83 to yield p3 and APP intracellular domain (AICD). In contrast, β-secretase commences APP cleavage in the latter pathway, generating soluble APP beta (sAPPβ) and C99, which contains the intact N-terminus of Aβ peptide. γ-secretase again completes this process, in this instance cleaving C99 to afford Aβ peptide of between 38 and 43 residues in length, and AICD. Most of the Aβ peptides produced are 40 residues in length, with the longer 42-residue variant making up a smaller proportion (Zhang et al., 2011). Despite accounting for a lesser percentage, the latter is understood to be more pathogenic compared to the former (Qiu et al., 2015).

Thus, given the potentially greater significance of Aβ_1−42_ in AD neuropathology, it would be most desirable to possess a ready means of access to this biologically relevant peptide in ample quantities, and perhaps more importantly, with purity levels that mirror the endogenous peptide. Indeed, chemical synthesis of Aβ peptide has been attempted by numerous research groups globally, predominantly employing 9-fluorenylmethoxycarbonyl (Fmoc)/*tert*-butyl (*t*Bu) solid phase peptide synthesis (SPPS) strategy, which was first introduced by Atherton et al. (1978). These past endeavors, however, have in general focussed on fragments of the peptide with established bioactivity (Wang et al., 2014), or the shorter Aβ_1−40_ variant (Choi et al., 2012).

While chemical syntheses of Aβ_1−42_ fragments are beneficial to the peptide research community, in that they have provided valuable structure-activity relationship (SAR) information with regards to key residues in the peptide that are involved in its aggregation and neurotoxicity properties, such data should be treated with care as these residues might behave in a distinct fashion when considered in context of the “full-length” Aβ_1−42_ peptide. The development of methodologies that enable preparation of Aβ_1−42_ in an efficient manner is therefore still very much in demand.

Routine preparation of Aβ_1−42_ is not typically undertaken owing to the “Aβ_1−42_ problem,” which is largely attributed to its propensity to aggregate both on resin during SPPS, as well as in solution. On-resin aggregation renders the free N-terminus inaccessible for coupling of subsequent amino acids in the sequence, resulting in either a truncated synthesis or an especially low crude recovery (Paradís-Bas et al., 2016). With regards to its characterization, purification of the peptide under conventional reverse phase-high performance liquid chromatography (RP-HPLC) conditions (acidic mobile phases, room temperature) yields an asymmetric, broad, and unresolved chromatographic peak, which is indicative of peptide aggregation. Thus, novel or improved protocols for preparation of this amyloidogenic peptide should be able to effectively mitigate these established issues, so as to afford the desired product in acceptable quantity and purity for further biological studies.

In this review, key publications concerning Aβ_1−42_ synthesis are critically discussed, providing readers with an insight into strategies that have been developed to overcome challenges associated with preparation of this “difficult peptide sequence.” For each publication reviewed herein, the synthetic protocol employed will be described in detail, and where the information was available, the overall yield and purity of the final product will be stated accordingly, as well as any related assays performed to provide satisfactory proof of bioequivalence.

## Burdick et al. (1992)

Burdick et al. is credited with the first reported synthesis of Aβ_1−42_ in 1992, utilizing continuous flow SPPS on a custom built synthesizer, which consisted of a Chontrol 4 outlet timer (Fisher Scientific), a back pressure regulator (Western Analytical), an FMI pump (Fluid Metering Inc.), and slider valves (Rainin). These elements were connected to a pressurized nitrogen gas source to actuate the valves. Furthermore, the synthesizer was fitted with a 5 ml sample loop (Rainin), and Omni columns and fittings (Omnifit).

**Scheme 1:**
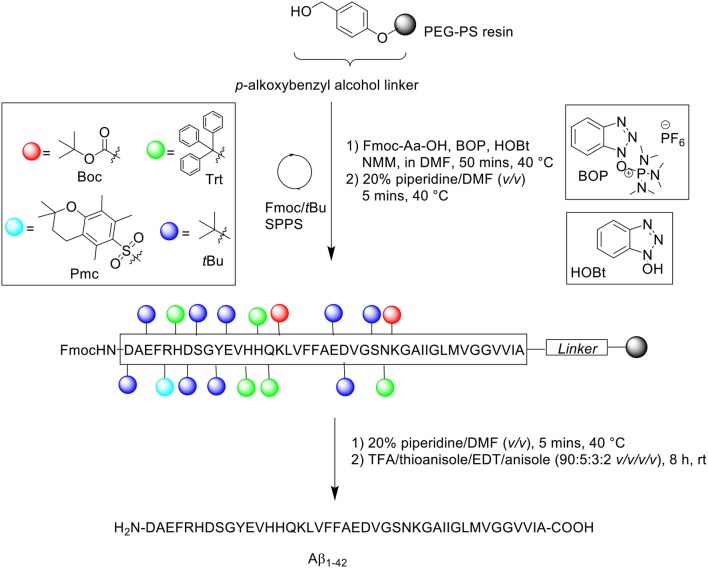
Fmoc/*t*Bu SPPS of Aβ_1−42_ by Burdick et al. (1992).

Peptide synthesis commenced on poly(ethylene) glycol-polystyrene (PEG-PS) resin (*loading not stated*), which was functionalized with a *p*-alkoxybenzyl alcohol linker (Scheme 1). The reactive side chains were protected as follows: arginine (Arg) residue was protected by 2,2,5,7,8-pentamethylchroman-6-sulfonyl (Pmc); asparagine (Asn), glutamine (Gln), and histidine (His) residues were protected by trityl; lysine (Lys) was protected by Boc; aspartic acid (Asp), glutamic acid (Glu), serine (Ser), tyrosine (Tyr), and threonine (Thr) residues were protected by *t*Bu (Burdick et al., 1992).

The reaction column was first immersed in a one-liter water bath at 40°C, washed with DMF at a flow rate of 5 mL/min for 1 min, and allowed to equilibrate gradually to room temperature as the reaction cycle progresses over time. Fmoc removal was achieved using 20% piperidine in DMF (*v/v*) for 5 min. Fmoc-amino acid couplings were afforded using Fmoc-Aa-OH (4 eq.), hydroxybenzotriazole (HOBt, 4 eq.), (benzotriazol-1-yloxy)tris(dimethylamino)phosphonium hexafluorophosphate (BOP, 4 eq.) in 5% *N*-methylmorpholine (NMM) in *N,N'*-dimethylformamide (DMF) (*v/v*) for 20 min, and repeated for a further 30 min with fresh reagents. A qualitative ninhydrin test was performed after each completed coupling cycle; coupling of Val12, His13, Val18, and Phe19 were reported by the authors to be incomplete, and consequently repeated. The completed peptide chain was cleaved from the resin using trifluoroacetic acid (TFA)/thioanisole/1,2-ethanedithiol (EDT)/anisole (90:5:3:2 *v/v/v/v*) at room temperature for 8 h. Crude peptides were dissolved in 88% formic acid (*v/v*) prior to purification on RP-HPLC using Vydac (Hesperia, CA) 214TP C4 column (10 μm, 2.2 × 2.5 cm, 300Å) at flow rate of 8 mL/min. A linear gradient between 0.1% TFA in water and 0.1% TFA in acetonitrile was employed over 55 min. Peak fractions were collected manually, lyophilized, and stored dry at −20°C until required. Peptide samples were characterized by electrospray ionization mass spectrometry (ESI-MS), amino acid sequencing by automated Edman degradation, and microscopy imaging. Additional assays were also undertaken to investigate the effects of pH and peptide concentration on peptide assembly. The results obtained show minimal to no sedimentation (as determined by γ-counting) at slightly basic pH and low peptide concentration, which suggested the need to employ basic buffers for purification of this peptide. No further bio-testing was attempted by the group.

**Scheme 2:**
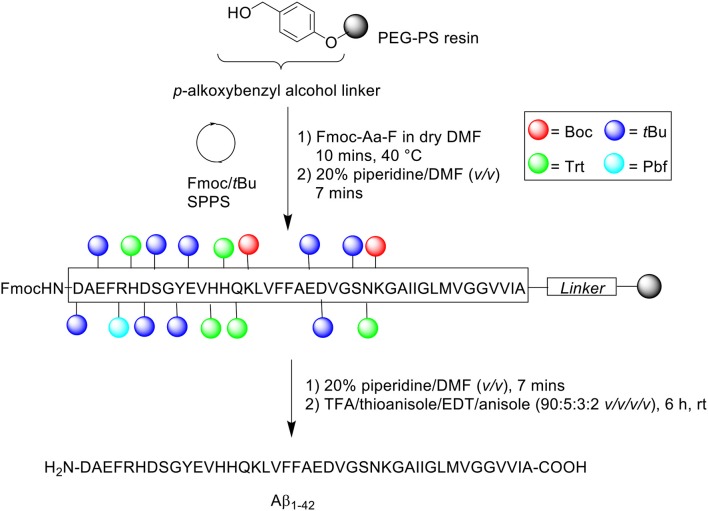
Aβ_1−42_ synthesized using preformed Fmoc-Aa-F by Milton et al. (1997).

A follow-up to this work was undertaken by Milton et al. (1997). The group utilized preformed Fmoc-aminoacyl fluorides (Fmoc-Aa-F) (Scheme 2) to facilitate the efficient synthesis of Aβ_1−42_ (Milton et al., 1997).

Previous studies have established the advantage conferred by using Fmoc-Aa-F building blocks over standard coupling reagents (Carpino et al., 1990; Wenschuh et al., 1995). Fmoc-Aa-F was prepared using two methods: dimethylaminosulfur trifluoride (DAST) and cyanuric fluoride. DAST was employed for the preparation of Fmoc-Aa-F of protected Ser, Val, Gly, Asn, Glu, and Met residues, while cyanuric fluoride was used to prepare Fmoc-Aa-F of protected Ala, Gln, Leu, Asp, Ile, Phe, Lys, and Tyr residues. Peptide synthesis commenced on a PEG-PS support (*loading not stated*), which was functionalized with a *p*-alkoxybenzyl ester to form an acid labile linker. For comparative purposes, three activation protocols were trialed: (1) BOP/HOBt/NMM – Fmoc-Aa-OH (4 eq.), BOP (4 eq.), HOBt (4 eq.) and NMM in DMF for 2 h at 40°C, (2) Preformed Fmoc-aminoacyl-fluorides – Fmoc-Aa-F (4 eq.) in dry DMF for 10 min at either 22 or 40°C, and (3) 1-[bis(dimethylamino)methylene]-1*H*-1,2,3-triazolo[4,5-*b*]pyridinium 3-oxide hexafluorophoshate (HATU)/*N,N*-diisopropylethylamine (DIPEA) – Fmoc-Aa-OH (4 eq.), HATU (4 eq.), DIPEA (8 eq.) in DMF for 1 h at 40°C. Fmoc removal was afforded using 20% piperidine in DMF (*v/v*) for 7 min at 40 or 55°C. The completed peptide chain was cleaved from the resin with TFA/thioanisole/EDT/anisole (90:5:3:2 *v/v/v/v*) at room temperature for 6 h. The TFA was then evaporated, and the remaining filtrate precipitated in cold diethyl ether, and stored overnight at −20°C. The frozen precipitate was sequentially washed with ether and allowed to dry *in vacuo*.

The crude peptide was purified by RP-HPLC using Vydac (Hesperia, CA) 214TP C4 column (10 μm, 2.2 × 2.5 cm, 300Å) at a flow rate of 8 mL/min using a linear gradient of 5–95% B over 60 min, where solvent B was 0.1% TFA in acetonitrile, whereas solvent A was 0.1% TFA in water. Fractions collected were immediately lyophilized, prior to analysis using a C4 column at a flow rate of 1 mL/mini. ESI-MS to determine presence of the desired product was undertaken by an external company (Peptidogenic Research, Livermore, CA). In the resulting HPLC spectrum, the major peak of Aβ_1−42_ synthesized with BOP/HOBt/NMM at 40°C for both acylation and deprotection steps was shown to not correspond to the desired product, which was identified to elute slightly later in time. Increasing Fmoc removal temperature to 55°C also yielded an identical spectrum. However, an increase in yield of the desired, but not major product, was noted, most likely due to a more “complete” removal of the temporary Fmoc protecting group. In contrast, the major peak of Aβ_1−42_ prepared using preformed Fmoc-Aa-F, carrying out the acylation and deprotection steps at 40 and 55°C, respectively, was shown to correspond to the desired product, thereby facilitating an easier purification process (Table 1). No further bio-testing was attempted by the group.

**Table 1:** Comparative Aβ_1−42_ yield from protocols trialed by Milton et al. (1997).

**Protocol**	**T_**acylation**_ (^**°**^C)**	**T_**deprotection**_ (^**°**^C)**	**Yield (%)**
BOP/HOBt/NMM	40	40	21
BOP/HOBt/NMM	40	55	22
Fmoc-Aa-F	22	40	23
Fmoc-Aa-F	40	40	25
Fmoc-Aa-F	40	55	28

*Yield was calculated as a percentage ratio between purified and crude weight*.

## Hendrix et al. (1992)

**Scheme 3:**
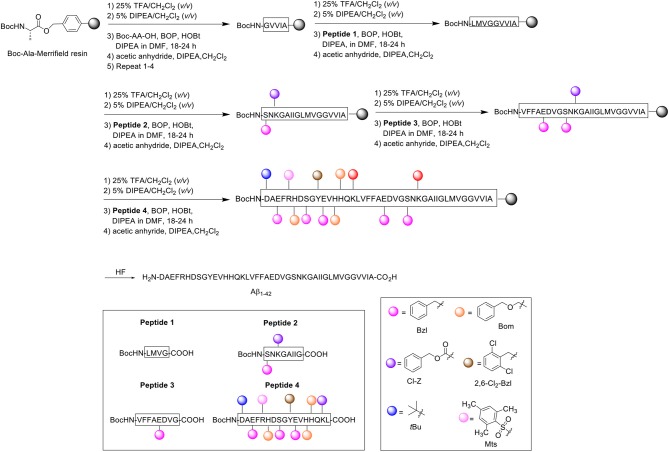
Boc/Bzl SPPS of Aβ_1−42_ by Hendrix et al. (1992).

During pioneering syntheses of Aβ_1−42_ by the Glabe laboratory, Hendrix et al. at the Massachusetts Institute of Technology, US also published their synthesis of the peptide (Scheme 3; Hendrix et al., 1992).

Noting one main disadvantage of stepwise SPPS for the preparation of relatively long peptides in general, which is the gradual accumulation of identical peptidic side products on resin, the group opted for a convergent approach instead, which they postulated would minimize the extent of side product formation. Using the *tert*-butyloxycarbonyl (Boc)/benzyl (Bzl) strategy, four Aβ_1−42_ fragments were synthesized and subsequently condensed on resin through their peptide backbone (Hendrix and Lansbury, 1992). All Boc-Aa-OH couplings were performed twice, followed by acetylation with acetic anhydride (10 eq.). The first fragment was synthesized on Kaiser oxime resin (Kaiser et al., 1989). Boc-protected leucine was bound to the resin and elongated to H-Y(2,6-Cl_2_-Bzl)E(Bzl)VH(Bom)H(Bom)QK(Cl-Z)L-resin. This fragment was subsequently reacted with Boc-D(*t*Bu)AE(Bzl)FR(Mts)H(Bom)DS(Bzl)G-OH (prepared with the same reagents and conditions described above) to afford the desired resin-bound **Peptide 4**, which was cleaved using low-high trifluoromethanesulfonic acid (TFMSA) /TFA protocol, purified by RP-HPLC, and characterized by amino acid analysis, ^1^H nuclear magnetic resonance (NMR), and fast atom bombardment mass spectrometry (FABMS).

The second fragment was prepared on Merrifield resin. Resin-bound Boc-protected alanine was first elongated to Boc-GVVIA-resin, after which **Peptide 1** (Boc-LMVG-CO_2_H) and **Peptide 2** (Boc-S(Bzl)NK(Cl-Z)GAIIG-CO_2_H) (1.2–1.5 eq., BOP activation at 23°C) were coupled sequentially on resin to form Boc-S(Bzl)NK(Cl-Z)GAIIGLMVGGVVIA-resin. **Peptide 3** (Boc-VFFAE(Bzl)DVG-CO_2_H) was coupled afterwards to complete the desired fragment. **Peptide 4** was coupled four successive times in the presence of BOP as an activating reagent to Boc-VFFAE(Bzl)D(Bzl)VGS(Bzl)NK(Cl-Z)GAIIGLMVGGVVIA-resin to complete the Aβ_1−42_ peptide chain, with each coupling step using less equivalents (2.3 eq. in total). The completed peptide chain was finally deprotected and cleaved off the resin using hydrogen fluoride.

Following analysis, it was identified that the crude product contained two truncated side products corresponding to Aβ_18−42_ and Aβ_26−42_. These low molecular weight impurities were fortunately separable using gel-filtration HPLC in 1,1,1,3,3,3-hexafluoro-2-propanol (HFIP), permitting high recovery of the desired product. The gel-purified material was then repurified by RP-HPLC to eliminate further minor side products, which included Aβ_1−37_ and benzylated Aβ_1−42_. The final purified product (>90% purity as determined by MS) was characterized by laser desorption MS and amino acid analysis employing Edman degeneration. The proposed method thus enabled the efficient synthesis of the peptide with minimal presence of side products. No further bio-testing was attempted by the group.

## Fukuda et al. (1999)

**Scheme 4:**
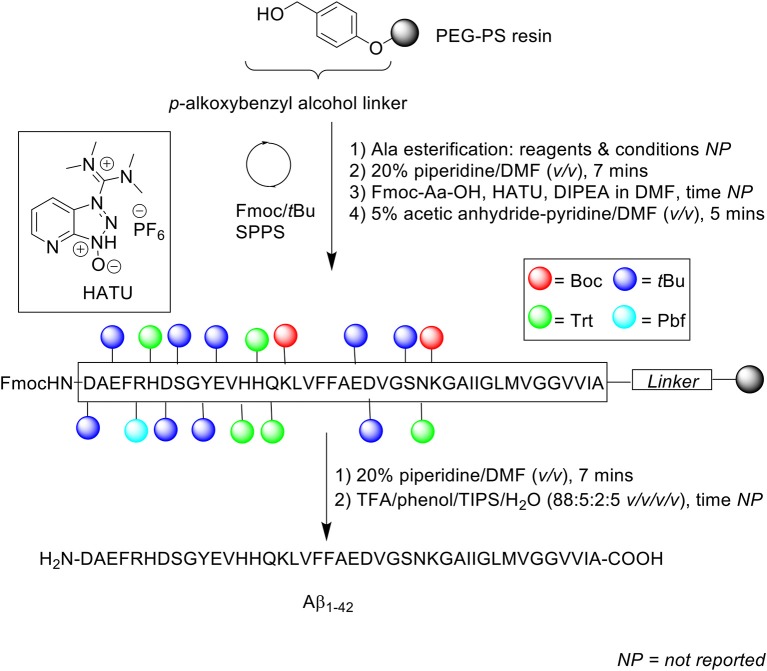
Fmoc/*t*Bu SPPS of Aβ_1−42_ by Fukuda et al. (1999).

Fukuda et al. reported on the successful synthesis of Aβ_1−42_ and its two isoaspartyl isomers at position 7 [Aβ_1−42_(isoAsp7)] and 23 [Aβ_1−42_(isoAsp23)] (Fukuda et al., 1999). Syntheses of all three peptides proceeded in a stepwise fashion using the Fmoc/*t*Bu SPPS strategy on PEG-PS resin (*loading not stated*) (Scheme 4).

Fmoc protecting group was deprotected using 20% piperidine in DMF (*v/v*) and Fmoc-amino acids (4 eq.) were single coupled in the presence of HATU (4 eq.) and DIPEA (8 eq.). For the isoaspartyl analogs, Fmoc-Asp-OtBu was used instead of Fmoc-Asp(OtBu)-OH at positions 7 and 23. Following each coupling step, any unreacted N-terminus was capped with 5% acetic anhydride-pyridine in DMF (*v/v*) for 5 min. The completed peptide chain was then cleaved off the resin using TFA/phenol/triisopropylsilane (TIPS)/water (88:5:2:5 *v/v/v/v*).

Given the propensity of the peptide to aggregate under acidic conditions, the lyophilized crude peptides were reconstituted in 0.1% ammonium hydroxide (NH_4_OH) prior to purification using RP-HPLC on Develosil 5 μm ODS-UG 140Å (150 × 4.6 mm) at 0.2 mL/min flow rate at room temperature. A linear gradient of 15–36% acetonitrile in 0.1% NH_4_OH was employed. Overall yields of each peptide were 10, 15, and 13% for Aβ_1−42_, Aβ_1−42_(isoAsp7), and Aβ_1−42_(isoAsp23), respectively, relative to starting crude material. The purity of the synthetic Aβ_1−42_ was subsequently compared with commercial Aβ_1−42_ (Bachem), using RP-HPLC, matrix assisted laser desorption ionization mass spectrometry (MALDI-TOF MS), and amino acid composition analysis. The results for all three analyses indicated that their Aβ_1−42_ had been synthesized at a higher purity compared to commercially obtained Aβ_1−42_. RP-HPLC analysis under alkaline conditions showed some impurities for commercial Aβ_1−42_, which was further confirmed by MALDI-TOF MS. In contrast, the group's Aβ_1−42_ yielded a single, sharp chromatographic peak with retention time *ca* 19 min. Amino acid composition analysis for their Aβ_1−42_ also produced a result that was closer to the theoretical value, in contrast to Bachem Aβ_1−42_. While purity of the final product was not reported by the authors, results obtained from thioflavin T (ThT) assay, transmission electron microscopy (TEM) imaging, and neurotoxicity assay against rat embryonic cortical neurons, carried out using their synthetic Aβ_1−42_, provided ample evidence that the synthesized Aβ_1−42_ is sufficiently bioequivalent to endogenous Aβ_1−42_.

## Tickler et al. (2001)

**Scheme 5:**
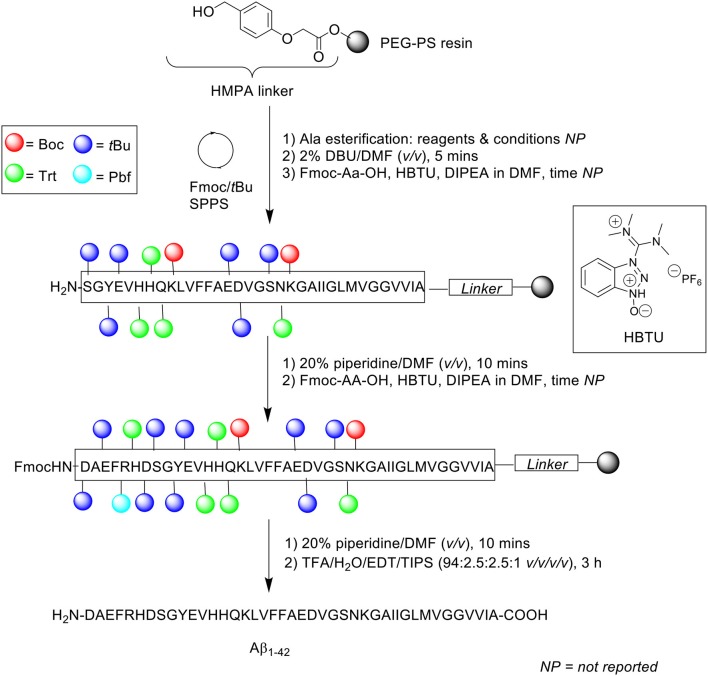
Fmoc/*t*Bu SPPS of Aβ_1−42_ using DBU as Fmoc deprotecting reagent by Tickler et al. (2001).

Another problematic issue for the synthesis of Aβ_1−42_ is the hydrophobicity of its C-terminal segment. In 2001, Tickler, Barrow, and Wade published on an improved preparation of this peptide, employing 1,8-diazabicyclo[5.4.0]undec-7-ene (DBU) as an Fmoc removal reagent (Scheme 5; Tickler et al., 2001).

Incomplete Fmoc removal is understood to be a factor that can affect overall synthetic efficiency. Thus, complete removal of temporary Fmoc protecting group from the N^α^-terminus of the amino acid is of paramount importance, as this facilitates efficient coupling of subsequent Fmoc-protected amino acids. For this purpose, the stronger base DBU was preferred to the conventionally used piperidine. Peptide synthesis was undertaken on PEG-PS resin (*loading not stated*) functionalized with 4-(hydroxymethyl)phenoxyacetic acid (HMPA) linker at 0.1 mmol scale. The Fmoc protecting group was removed using 2% DBU in DMF (*v/v*) for 5 min, up until residue Ser8, after which 20% piperidine in DMF (*v/v*) was employed to prevent aspartamide formation at residue Asp7, an established side reaction observed in the chemical synthesis of Aβ_1−42_. Consequently, deprotection time for subsequent Fmoc-amino acid residues was also extended to 10 min. Fmoc-amino acids were coupled in the presence of 3-[bis(dimethylamino)methyliumyl]-3*H*-benzotriazol-1-oxide hexafluorophosphate (HBTU) and DIPEA in DMF. Cleavage of the completed peptide chain was accomplished with TFA/water/EDT/TIPS (94:2.5:2.5:1 *v/v/v/v*) for 3 h. The TFA filtrate was then concentrated *in vacuo*, precipitated in cold ether, reconstituted in water/acetonitrile/TFA (90:10:0.1 or 80:20:0.1 *v/v/v*), and recovered by lyophilization (40% yield based on 0.1 mmol PEG-PS resin loading).

Characterization of the crude peptide was performed using RP-HPLC on a Vydac (Hesperia, USA) C4 analytical column (4.6 × 250 mm, 5 μm) at 1 mL/min flow rate at 60°C. A linear gradient of 15–50% B was employed over 30 min, where solvent A was 10 mM ammonium bicarbonate (NH_4_CO_3_) and solvent B was acetonitrile. The resulting HPLC spectrum yielded predominantly a single chromatographic peak with a retention time *ca* 19 min, which mass was confirmed by MALDI-TOF MS to correspond to the desired product. Purification of the crude product was undertaken using RP-HPLC, affording pure Aβ_1−42_ at 17% yield relative to purified crude weight. The purity of the final product, however, was not reported by the authors. Furthermore, employment of bicarbonate-based buffers, while shown herein to be beneficial with regards to purification of their Aβ_1−42_, still required further investigation, particularly to probe the bioequivalence of the purified peptide. This was unfortunately not attempted by the group. Regardless, the employment of DBU in the synthesis of Aβ_1−42_ to improve Fmoc removal efficiency, especially at its hydrophobic C-terminal region, was demonstrated to afford a crude product of higher quality, thus translating to a relatively easier purification process.

## Carpino et al. (2004)

**Scheme 6:**
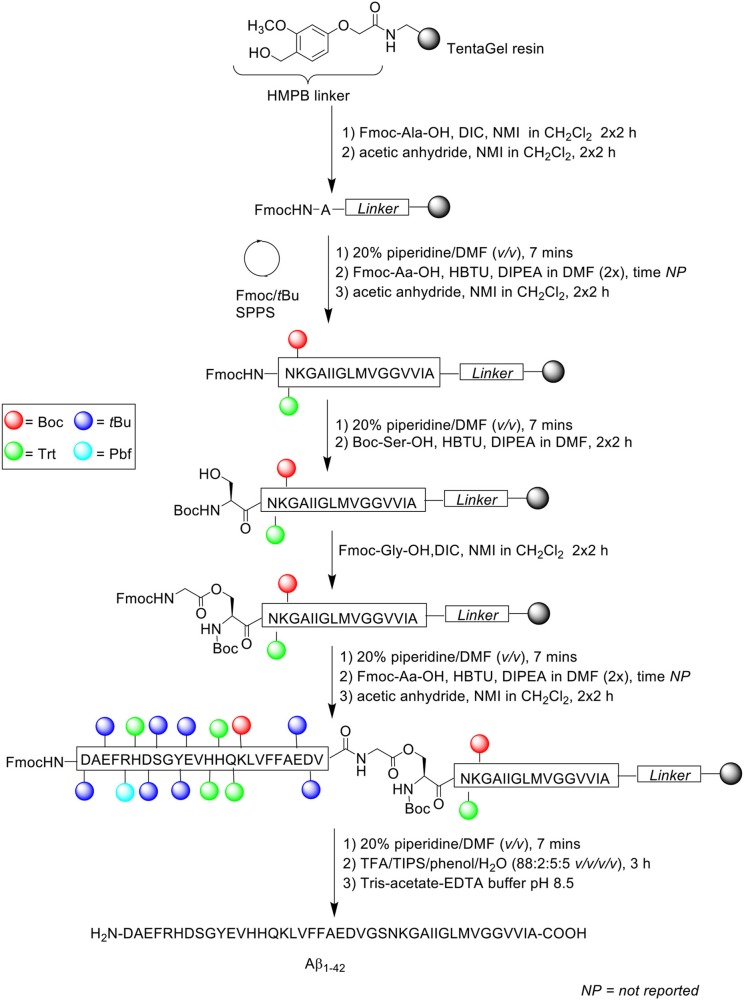
Fmoc/*t*Bu SPPS of Aβ_1−42_ using depsipeptide methodology by Carpino et al. (2004).

In the attempt to address the prevailing issue of Aβ_1−42_ aggregation on resin, Carpino et al. incorporated the depsipeptide method in their synthesis of Aβ_1−42_ (Scheme 6; Carpino et al., 2004). It is thought that the introduction of depsipeptides, a class of peptidic compounds in which the peptide bonds have been substituted with amide bonds, may restrict aggregation phenomena during synthesis (Coin, 2010).

Instead of synthesizing the linear peptide, its water-soluble *O*-acyl isopeptide was prepared instead, selecting Ser26 as the residue to perform *O*-acylation on, as the Gly25 residue does not epimerize during activation to form the ester bond. Peptide chain was subsequently elongated as normal thereafter. Following cleavage from the resin, the *O*-acyl isopeptide can be rearranged to yield the native peptide by treatment in aqueous buffer at pH 8.

Peptide synthesis commenced on TentaGel resin (0.25 mmol/g loading) functionalized with 4-(4-hydroxymethyl-3-methoxyphenoxy)butyric acid (HMPB) linker at 0.125 mmol scale. Fmoc-Ala-OH (1 mmol) was double coupled manually on resin in the presence of *N,N'*-diisopropylcarbodiimide (DIC) and *N*-methylimidazole (NMI) in dichloromethane (CH_2_Cl_2_) for 2 h each time. Any free amine at the N-terminus was capped twice with acetic anhydride/NMI (0.75 mmol) in CH_2_Cl_2_ for 2 h each time. Fmoc-amino acids were then double coupled up to residue Asn27 using Fmoc-Aa-OH, HBTU, and DIPEA in DMF. The Fmoc protecting group on Asn27 was removed prior to double coupling of Boc-Ser-OH using HBTU and DIPEA in DMF for 2 h each time. Resin-bound Ser26 residue was then *O*-acylated with Fmoc-Gly-OH/DIC/NMI and capped under the conditions stated above. The peptide sequence was then elongated to completion, cleaved off resin with TFA/TIPS/phenol/water (88:2:5:5 *v/v/v/v*) for 3 h, and recovered by lyophilization. Rearrangement of the *O*-acyl isopeptide to native Aβ_1−42_ was achieved by treatment with Tris-acetate-ethylenediaminetetraacetic acid (EDTA) buffer (pH 8.5). The overall yield and purity of the final peptide were not reported.

A significant by-product corresponding to Aβ_26−42_ was detected in the resulting crude HPLC profile, which was postulated to be due to incomplete *O*-acylation. Further bio-testing was also not undertaken by the group.

## Kim et al. (2004)

**Scheme 7:**
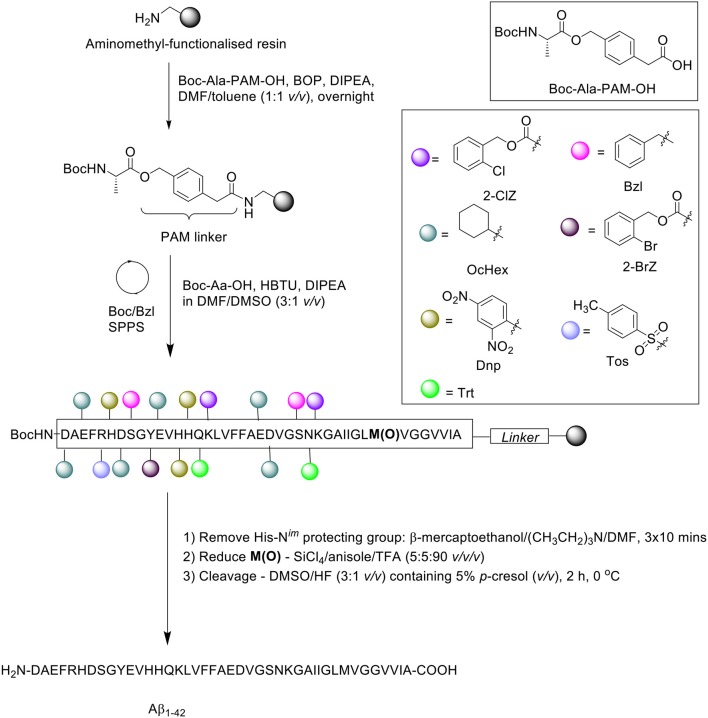
Boc/Bzl SPPS of Aβ_1−42_ with oxidized Met and DMSO as coupling co-solvent by Kim et al. (2004).

In the same year, Kim et al. presented an optimized coupling reaction for the efficient synthesis of Aβ_1−42_ (Kim et al., 2004), which capitalized on the known disaggregating role of the single methionine residue in the sequence (Scheme 7). Intentional incorporation of oxidized methionine in the peptide synthesis consequently permitted the use of dimethylsulfoxide (DMSO) as co-solvent in all coupling reactions performed, which would normally oxidize methionine to its sulfoxide derivative. The authors postulated that DMSO would produce an additional disaggregating effect in the synthesis.

Peptide synthesis commenced on aminomethyl-functionalized resin (1.0 mmol/g loading) at 0.2 mmol scale, employing the Boc/Bzl SPPS strategy. Two syntheses were performed in parallel for comparative purposes. Firstly, the alanine residue was coupled on resin using preformed Boc-Ala-phenylacetamidomethyl (PAM)-OH (1.5 eq.) in the presence of BOP (2 eq.) and DIPEA (3 eq.). In the first synthesis, Met35 was incorporated in its sulfoxide form, Met(O)-35, whereas the native methionine residue was used for the second synthesis. Boc-amino acid couplings were carried out in the presence of DMF/DMSO (3:1 *v/v*). Following completion of the peptide chain, the His-N^*im*^-Dnp protecting group was removed using a cocktail of β-mercaptoethanol and triethylamine in DMF for 3 × 10 min, and the sulfoxide moiety reduced using a cocktail of silicon tetrachloride (SiCl_4_)/anisole/TFA (5:5:90 *v/v/v*) for 15 min. The peptide was then recovered from the resin using DMSO/HF (3:1 *v/v*) with 5% *p*-cresol (*v/v*) for 2 h at 0°C, and lyophilized.

RP-HPLC analysis was performed on a Vydac 219TP5415 diphenyl column (300Å, 4.6 × 150 mm, 5 μm), using a linear gradient of solvent A (0.1% TFA in water) and B (0.09% TFA in acetonitrile). The eluted chromatographic peak (retention time 19.3 min) was identified by MALDI-TOF MS to match the desired product, although the yield and purity of the final product was not reported by the group. Furthermore, one limitation of this method, as acknowledged by the authors, is the presence of deletion products corresponding to deletion of one or two phenylalanine residues in the sequence. No further bio-testing was attempted by the group.

## Sohma et al. (2005)

Sohma et al. also implemented the “*O*-acyl isopeptide method” in their synthesis of Aβ_1−42_ (Sohma et al., 2005). For comparative purposes, Aβ_1−42_ was also synthesized in a linear fashion on 2-chlorotrityl chloride resin (*loading not stated*) at 0.3 mmol scale. In the synthesis of the linear peptide, Fmoc-Ala-OH was coupled for 2.5 h in the presence of DIPEA in 1-ethyl-3-(3-dimethylaminopropyl)carbodiimide (EDC) under argon atmosphere, followed by capping with methanol in the presence of DIPEA in DMF for 20 min. The resin bed was afterwards washed sequentially with DMF, DMF/water (1:1 *v/v*), chloroform (CHCl_3_), methanol, and then dried *in vacuo*. Alanine loading was photometrically quantified based on liberation of Fmoc chromophore following treatment with 50% piperidine in DMF (*v/v*) for 30 min at 37°C. Fmoc removal was afforded with 20% piperidine in DMF (*v/v*) for 20 min, and subsequent Fmoc-amino acid residues were manually coupled using DIC/HOBt in DMF for 2 h. The completed peptide chain was cleaved off the resin using TFA/*m*-cresol/thioanisole/water (92.5:2.5:2.5:2.5 *v/v/v/v*) for 90 min, the filtrate concentrated *in vacuo*, precipitated with cold ether, reconstituted in water, and recovered by lyophilization.

A sample of the crude product was then dissolved in TFA/water (2:1 *v/v*) in the presence of ammonium iodide (NH_4_I) and dimethyl sulfide (CH_3_)_2_S, then stood for 1 h at 0°C to reduce any oxidized methionine residues. The mixture was then concentrated *in vacuo*, dissolved in HFIP, and filtered using a 0.46 μm filter unit, before purification using preparative HPLC on a C18 column (4.6 × 150 mm; YMC Pack ODS AM302) at 40°C. A linear gradient of 0–100% acetonitrile in 0.1% aqueous TFA was employed over 40 min at a flow rate of 5 mL/min. Peak fractions were collected and immediately lyophilized to afford pure Aβ_1−42_ (overall yield of 7.2% relative to starting crude material, purity >94%). MALDI-TOF MS and RP-HPLC analyses of collected fractions confirmed the presence of the desired product, which had a retention time that matched commercial Aβ_1−42_.

**Scheme 8:**
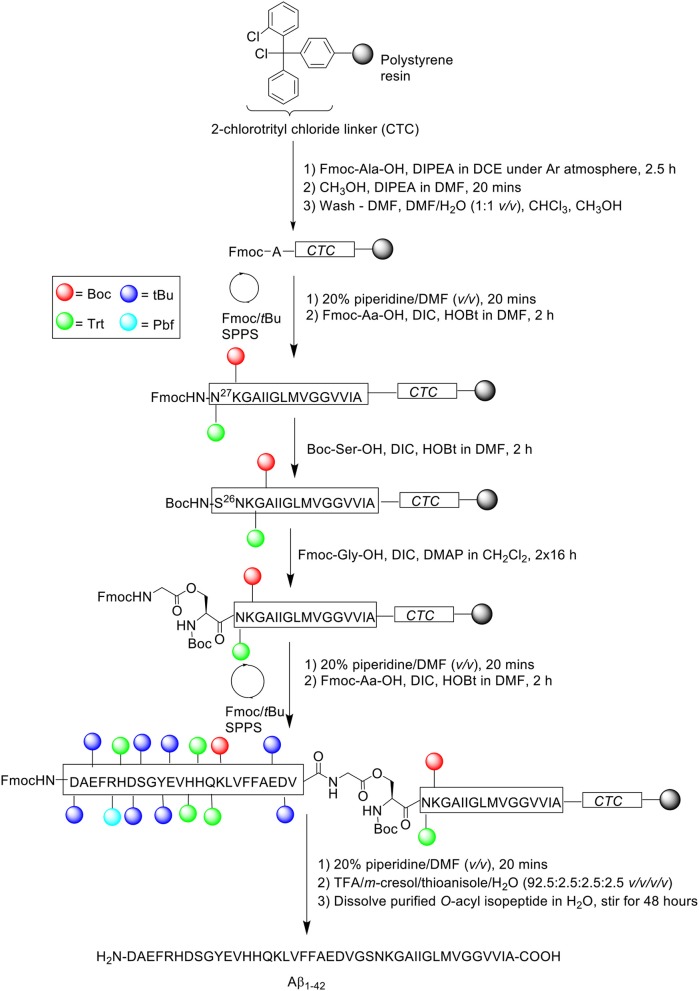
Fmoc/*t*Bu SPPS of '26-O-acyl-isoAβ_1−42_' by Sohma et al. (2005).

Synthesis of “26-*O*-acyl-isoAβ_1−42_” was subsequently attempted. Fmoc-protected Aβ_27−42_ was assembled on 2-chlorotrityl chloride resin at 0.1 mmol scale, employing the same reagents and conditions as described above (Scheme 8).

Following completion of the peptide fragment, Boc-Ser-OH was coupled in the presence of DIC and HOBt in DMF for 2 h, followed by double coupling of Fmoc-Gly-OH using DIC and 4-dimethylaminopyridine (DMAP) in CH_2_Cl_2_ for 16 h each time. Peptide assembly was then continued to afford the completed *O*-acyl isopeptide chain, which was cleaved off the resin and recovered by lyophilization. The crude peptide was also pre-treated with NH_4_I/(CH_3_)_2_S before purification, which afforded pure *O*-acyl isopeptide at 33.6% yield relative to purified crude weight and >96% purity. The purified peptide was subsequently dissolved in water and stirred for 48 h to induce *O*- to *N*-acyl transfer reaction, yielding Aβ_1−42_ quantitatively. RP-HPLC profile and MALDI-TOF MS of Aβ_1−42_ synthesized using this method was again shown to be identical to that of commercial Aβ_1−42_. No further bio-testing was attempted by the group.

## GarcÍa-MartÍn et al. (2006)

**Scheme 9:**
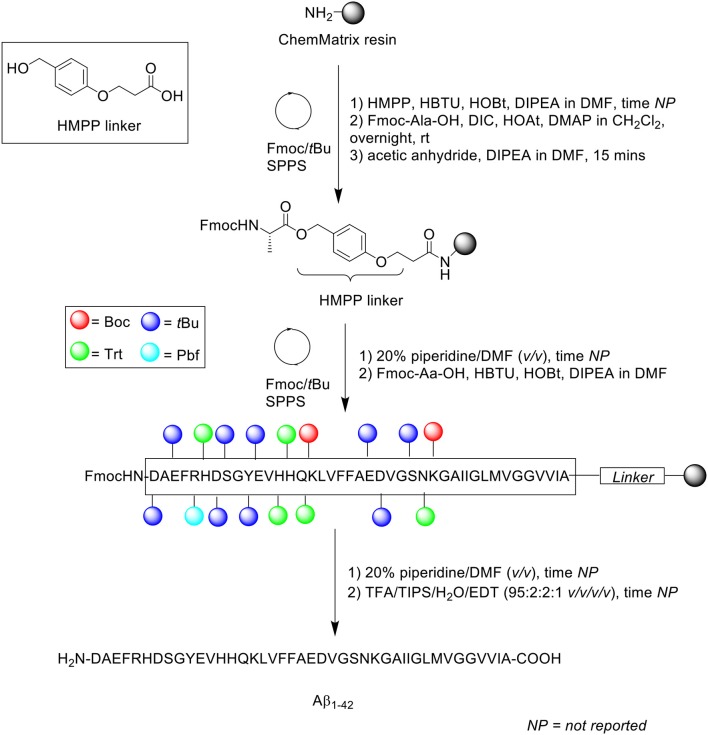
Fmoc/*t*Bu SPPS of Aβ_1−42_ on ChemMatrix resin by García-Martín et al. (2006).

The propensity of Aβ_1−42_ to aggregate on resin during SPPS necessitates some considerations with regards to the choice of solid support. Particularly, the use of resins with a low degree of substitution might be more appropriate for the efficient preparation of long, hydrophobic peptides in general, as lower loading translates to lesser steric interference on resin. In 2006, García-Martín et al. introduced ChemMatrix resin as a novel, solid support for SPPS (García-Martín et al., 2006). ChemMatrix is a totally PEG-based resin which consists of only primary ether bonds, which renders it chemically stable. It also assumes a free-flowing form upon drying *in vacuo*, and is a preferable choice over PS-based resins in the synthesis of “difficult peptide sequences.” Firstly, the physical properties of ChemMatrix was contrasted with PS. The former was shown to swell in most solvents, including especially polar ones such as acetonitrile, DMSO, and methanol; none of the three can swell PS resins satisfactorily. More importantly, ChemMatrix swelled better than PS in DMF and CH_2_Cl_2_, and also exhibited higher chemical stability toward acid or base treatment, with the sole exception of strong Lewis acids. Microscopic analysis of ChemMatrix beads following shrinking and swelling by various solvents demonstrated no notable structural deterioration, which suggested that it was unaffected by osmotic stress. Subsequently, this resin was employed in the synthesis of four complex peptides: a decameric model peptide consisting of oligo(aminoacyl) sequences, 38-amino acid long synthetic vaccine Bacuma, polyarginine peptide, and Aβ_1−42_ peptide (Scheme 9).

Aβ_1−42_ synthesis was carried out on ChemMatrix resin (*loading not stated*) at 0.07 mmol scale in a stepwise fashion. The acid labile 3-(4-hydroxymethylphenoxy)propionic acid (HMPP) linker was coupled on resin manually with HBTU, HOBt, and DIPEA in DMF, followed by first residue attachment (Fmoc-L-Ala-OH) using DMAP in CH_2_Cl_2_ overnight at room temperature. Despite a prolonged reaction time, free hydroxyl groups were still present, as determined by the 4(4-nitrobenzyl)pyridine test. Consequently, the alanine coupling step was repeated for a further 2 h with fresh reagents. Acetylation of free N-terminus group was afforded using acetic anhydride/DIPEA in DMF for 15 min, followed by automated elongation to generate the desired peptide chain, which was cleaved off the resin using TFA/TIPS/water/EDT (95:2:2:1 *v/v/v/v*) for 90 min.

Following recovery by lyophilization, the peptide was monomerized in neat TFA, concentrated *in vacuo*, and dissolved in HFIP to retain the disaggregated state. RP-HPLC analysis was undertaken using a C8 column (size not reported; flow rate 1 mL/min) at 60°C. A linear gradient between 0.1% TFA in water and 0.1% TFA in acetonitrile was employed over 15 min. The resulting HPLC spectrum showed a single major peak with a retention time of 9.3 min and an estimated purity of 91%, which was confirmed by MALDI-TOF MS to correspond to the desired product. Unfortunately, the yield was not reported, and no further bio-testing was carried out by the group. Furthermore, it was not explicitly stated whether purification of the crude product was attempted in this study, although the relatively high purity percentage suggested this was the case.

## Zarándi et al. (2007)

**Scheme 10:**
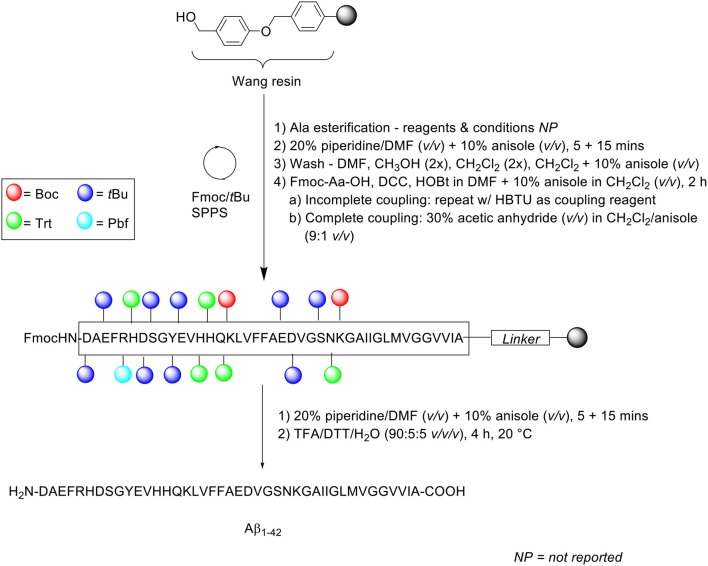
Fmoc/*t*Bu SPPS of Aβ_1−42_ using anisole as co-solvent by Zarándi et al. (2007).

Zarándi et al. introduced anisole as a relatively cheap and simple co-solvent for both deprotection and coupling steps in their Fmoc/*t*Bu SPPS of Aβ_1−42_ (Scheme 10; Zarándi et al., 2007). It was postulated that the use of anisole would improve the purity and yield of crude Aβ_1−42_ by preventing its aggregation during synthesis.

Peptide synthesis commenced on Wang resin (0.41 mmol/g loading) at 0.25 mmol scale. Fmoc removal was performed twice with 20% piperidine in DMF (*v/v*) containing 10% anisole (*v/v*) for 5 and 15 min. Following each deprotection step, the resin bed was washed sequentially with DMF, methanol (twice), CH_2_Cl_2_ (twice), and a final wash with 10% anisole in CH_2_Cl_2_ (*v/v*). Fmoc-amino acids were coupled in the presence of *N,N'*-dicyclohexylcarbodiimide (DCC) and HOBt in DMF, which was further diluted with 10% anisole in CH_2_Cl_2_ (*v/v*) for 2 h. The completeness of the reaction was qualitatively evaluated using the ninhydrin test: in the case of an incomplete reaction, the coupling step was repeated with HBTU as a coupling reagent, otherwise, acetylation was carried out using 30% acetic anhydride (*v/v*) in CH_2_Cl_2_/anisole (9:1 *v/v*) prior to subsequent deprotection-coupling cycles. The completed peptide chain was cleaved off the resin with TFA/dithiothreitol (DTT)/water (90:5:5 *v/v/v*) at 20°C for 4 h.

Following cleavage, the TFA filtrate was diluted with 0.1% TFA in acetonitrile to a final acetonitrile concentration of 30% (*v/v*), and afterwards loaded into a preparative HPLC column. Purification of the crude peptide was carried out on a PrepPak® Cartridge 47 x 300 mm (column no. M23582) Bakbond WP C4 15 μ Packing column at a flow rate of 80 mL/min. A linear gradient of 30–70% B was employed, where solvent B was 0.1% TFA in acetonitrile/water (4:1 *v/v*) and solvent A was 0.1% TFA in water. The collected fractions were analyzed by analytical RP-HPLC, ESI-MS, and amino acid composition. RP-HPLC analysis was undertaken using a Phenomenex (Jupiter) C4 column (250 × 4.6 mm, 300Å, 5 μm) at a flow rate of 0.2 mL/min. A linear gradient of 30–90% B was employed, using the same solvent system as that for peptide purification. The eluted chromatographic peak was also confirmed by ESI-MS and amino acid analysis to correspond to the desired product, although neither its yield nor purity were reported by the authors. The 3-(4,5-dimethylthiazol-2-yl)-2,5-diphenyltetrazolium bromide (MTT) assay was subsequently carried out on a SH-SY5Y neuroblastoma cell line, which was incubated in the presence of synthetic Aβ_1−42_. The results obtained showed an expected reduction in cellular viability by ~ 40%, compared to untreated control cells, which provided proof of bioequivalence.

## Bacsa et al. (2010)

**Scheme 11:**
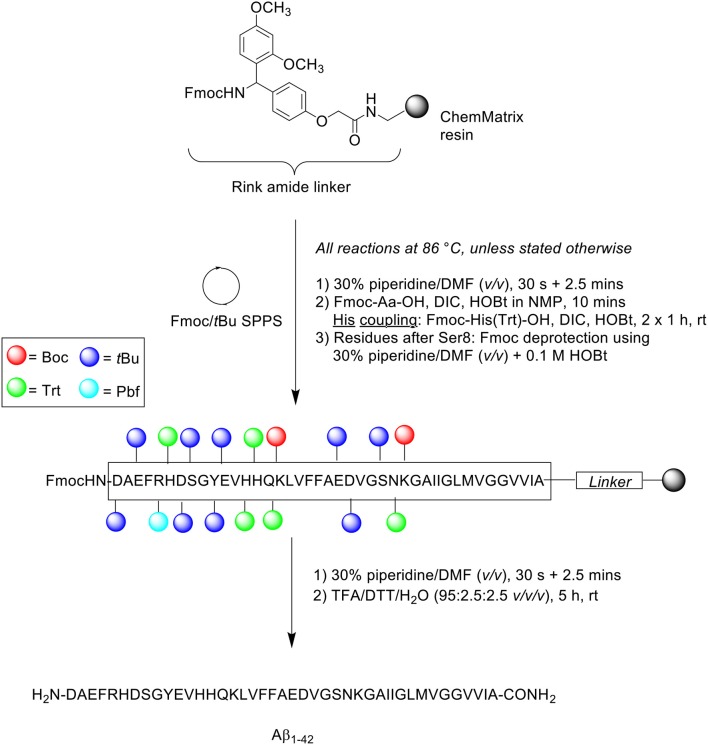
Microwave-assisted Fmoc/*t*Bu SPPS of Aβ_1−42_ by Bacsa et al. (2010).

Owing to its length, and the use of extended coupling times, preparation of synthetic Aβ_1−42_ typically takes up to 48 h. Bacsa et al. thus attempted to synthesize this peptide using microwave-assisted Fmoc/*t*Bu SPPS to accelerate the coupling and deprotection steps, affording the final peptide in a shorter overall preparation time (Scheme 11; Bacsa et al., 2010) Microwave-assisted synthesis is thought to perturb intermolecular hydrogen bonding between neighboring β-sheets, a key interaction in peptide aggregation (Paradís-Bas et al., 2016).

Peptide synthesis was undertaken on Rink amide ChemMatrix resin (0.50 mmol/g loading) at 0.075 mmol scale. All reactions were performed at 86°C, unless indicated otherwise. Fmoc removal was afforded in two steps using 30% piperidine in DMF (*v/v*), first for 30 s and then 2.5 min. Fmoc-amino acids were single coupled in the presence of DIC and HOBt in *N*-methyl-2-pyrrolidone (NMP) for 10 min. Coupling of the three sensitive histidine residues had to be performed at room temperature in order to prevent racemization. This step was performed twice for 60 min each time to ensure complete coupling, using Fmoc-His(Trt)-OH and DIC/HOBt. The coupling cocktail was allowed to pre-activate for 2 min prior to addition to resin bed. Fmoc removal of residues after Ser8 was achieved using 30% piperidine in DMF (*v/v*) containing 0.1 M HOBt to prevent aspartamide formation at residue Asp7. The completed peptide chain was cleaved using TFA/DTT/water (95:2.5:2.5 *v/v/v*) for 5 h at ambient temperature. The TFA filtrate was then concentrated *in vacuo*, precipitated with cold ether, and recovered by lyophilization to afford crude peptide at 78% yield. This was immediately stored at −20°C to avoid methionine oxidation and further aggregation of the peptide.

A sample of the crude peptide was dissolved in HFIP prior to RP-HPLC analysis using an analytical Phenomenex Jupiter C4 column (250 × 4.6 mm, 10 μm) at 60°C. A linear gradient of 30–100% B was applied over 45 min at a flow rate of 4 mL/min, where solvent B was 0.1% TFA in CH_3_CN and solvent A was 0.1% TFA in H_2_O. The resulting chromatogram showed a single major peak with a retention time of 4.14 min, the mass of which was confirmed by MALDI-TOF MS to correspond to the desired product. Furthermore, this result was also corroborated by standard amino acid analysis and comparison of the ^1^H NMR spectrum of the crude peptide with previously published data. The group also undertook Aβ_1−42_ synthesis using conventional heating at the same temperature, which interestingly also yielded similar results to that obtained from microwave-assisted synthesis. The neurotoxicity of the synthesized peptides were examined *in vitro* by conducting an MTT assay on a SH-SY5Y cell line, which produced identical results with respect to cellular viability. Taken together, these results indicated that rapid synthesis of Aβ_1−42_ can be achieved with microwave-assisted Fmoc/*t*Bu SPPS, and that Aβ_1−42_ synthesized using this method is sufficiently bioequivalent. The latter conclusion is particularly important as the use of microwave energy in peptide synthesis has been associated with peptide backbone de-aggregation, due to its direct interactions with the generated electric field.

## Collins et al. (2014)

In the attempt to further shorten the preparation time and improve yield of Aβ_1−42_, Collins et al. developed the high efficiency SPPS (HE-SPPS) methodology, which was successfully implemented in the synthesis of several complex peptides, including Aβ_1−42_ (Collins et al., 2014). Firstly, two conventional, non-microwave-assisted syntheses of Aβ_1−42_ were trialed at 0.1 mmol scale on PAL-PEG-PS resin (0.16 mmol/g loading) to establish a baseline purity level. In the first synthesis, Fmoc removal was performed twice for 5 and 10 min. Fmoc-amino acid (5 eq.) coupling was achieved in the presence of DIC (5 eq.) and Oxyma (5 eq.) in DMF for 60 min. The completed peptide chain was cleaved with TFA/TIPS/water/2,2′-(ethylenedioxy)diethanethiol (DODT) (*v/v/v/v*) and recovered by lyophilization to afford the crude peptide at 85% yield and 56% purity. In the second synthesis, Fmoc removal was also performed twice, but at shorter times of 0.5 and 3 min. Fmoc-Aa-OH (5 eq.) was coupled using 2-(6-chloro-1*H*-benzotriazole-1-yl)-1,1,3,3-tetramethylaminium hexafluorophosphate (HCTU, 5 eq.) in DMF and DIPEA (10 eq.) in NMP for only 5 min. The relatively short coupling time may provide an explanation to the staggeringly low crude purity of 14%, even though the yield was 72%. Aβ_1−42_ synthesis was also attempted using microwave-assisted SPPS. Fmoc removal was afforded by 20% piperidine (*v/v*) containing 0.1 M Oxyma. TFA cleavage was performed for 30 min at 38°C, and other conditions remained as stated above. This afforded the crude peptide at 87% yield and 67% purity.

Optimizations of the microwave protocol was subsequently undertaken as follows: first, microwave conditions for deprotection and coupling steps were assessed. The group observed that the duration of these steps can be significantly shortened through utilization of a higher microwave power, whilst maintaining a high temperature within the reaction vessel. This setup allowed the vessel to reach 90°C (T_max_ = 92°C) in just 20 s, as monitored using a fiber optic probe. Consequently, it was possible for deprotection and coupling steps to be completed in only 1 and 2 min, respectively. Next, the washing step was assessed. It was noted that following completion of each coupling reaction, the resin bed retained a residual temperature of 50°C, which would permit diffusion at a higher rate, and thus more efficient washing steps, employing less reagent volumes and shorter time. Furthermore, the authors reasoned that post-coupling washes are not necessary, as the inherent protection afforded by SPPS meant that uncoupled, activated Fmoc-amino acids in solution would be dissolved by the large excess of base introduced in the subsequent deprotection step. Lastly, an assessment of reagents used for Fmoc removal was undertaken. Due to the classification of piperidine as a controlled substance, piperazine has often been considered as an alternative Fmoc deblocking reagents for both conventional and microwave synthesis. However, one major limitation of this reagent is that it can only reach a maximum concentration of 6% (*v/v*) when diluted in either DMF or NMP. A novel solvent system was thus conceived, which was aimed to increase piperazine concentration without bearing negative effects on solubility of the resin-bound peptide. Dilution of piperazine in ethanol/NMP (1:9 *v/v*) successfully elevated its maximum concentration to 10% (*v/v*), which was shown to be equally effective to 20% piperidine (*v/v*) with regards to its deprotecting capacity. These optimizations were implemented in the synthesis of Aβ_1−42_. Aspartamide formation, an established side reaction in the synthetic process, was reasonably minimized using these reaction conditions. The proposed method enabled the efficient preparation of Aβ_1−42_ in just under 4 h, which is remarkably fast for a peptide of this length. All samples were analyzed by ultra-high performance liquid chromatography (UPLC) using Acquity UPLC BEH C18 column (1.7 mm and 2.1 × 100 mm). A crude 87% yield was obtained after lyophilization with 10% acetic acid (*v/v*) at 72% purity. Furthermore, the overall protocol also minimized chemical waste by approximately 90%, which is a forward step toward sustainability in peptide synthesis. Unfortunately, no further bio-testing was attempted by the group.

## Chemuru et al. (2014)

**Scheme 12:**
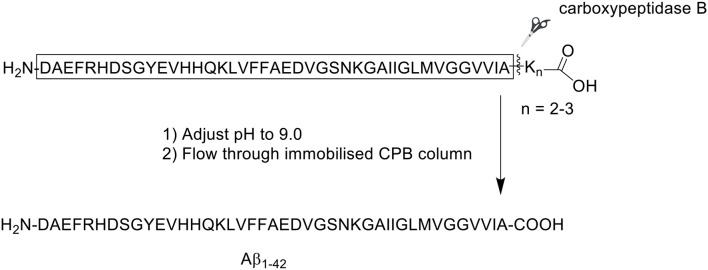
Enzyme-mediated separation of lysine tag from Aβ_1−42_ peptide by Chemuru et al. (2014).

An alternative approach by which the efficient synthesis of Aβ_1−42_ may be achieved is through adjustment of its solubility properties, which dictate how the peptide behaves in solution. Given the presence of hydrophobic residues proximal to its C-terminus, Aβ_1−42_ expectedly commences aggregation almost immediately following reconstitution in conventional solvents such as acetonitrile. In 2014, Chemuru et al. reported on the ready separation of Aβ_1−42_ pre-synthesized with a C-terminal solubilizing tag consisting of two or three lysine residues (Scheme 12; Chemuru et al., 2014) Peptides were synthesized using Fmoc/*t*Bu SPPS on PEG-PS resin (*loading not stated)*. Fmoc-protected amino acids were double coupled in the presence of HBTU and NMM in DMF.

The crude product was first dissolved in 50% aqueous formic acid (*v/v*) prior to purification by RP-HPLC on an Agilent Zorbax SB-C3 column (9.4 × 250 mm, 5 μm). A linear gradient of 30–60% B was employed, where solvent B was 0.05% TFA in acetonitrile, and solvent A was 0.05% TFA in water. For comparative purposes, the group also procured synthetic wild-type Aβ_1−42_. Purification of native Aβ_1−42_ was attempted at both room temperature and 65°C, whereas Aβ_1−42_ tagged with lysine residues was readily purified at room temperature. RP-HPLC analysis of Aβ_1−42_ at room temperature employing the solvent system described above yielded the expected asymmetrical, broad, and unresolved chromatographic peak. The yield and purity of Aβ_1−42_ were 2.7 and 64.9%, respectively. Repeating the purification process at 65°C resolved the peak, affording the desired product in 6.4% yield with 80.6% purity.

Following purification, the C-terminal lysine residues were removed using a carboxypeptidase B (CPB) agarose column, which was equilibrated to room temperature and washed at least five times with Tris-buffered saline (TBS, pH 9.0) prior to use. Purified Aβ_1−42_-Lys tail peptide was flowed through the column at approximately 0.2 mL/min. The column was then washed with TBS. One-milliliter fractions were collected from both peptide load and column wash, then analyzed by liquid chromatography-mass spectrometry (LC-MS) to identify peptide-containing fractions, and confirm complete removal of the Lys residues. Fractions containing the desired product were pooled, the pH adjusted to 2.0, loaded onto RP-HPLC column, and repurified using the conditions described above. The collected fractions were assessed for purity, then pooled and lyophilized, affording pure Aβ_1−42_. For Aβ_1−42_ synthesized with two C-terminal lysine residues (Aβ_1−42_K_2_), the yield and purity of the final compound was 6.2 and 89.7%, respectively. Interestingly, the yield of Aβ_1−42_ synthesized with three C-terminus lysine residues (Aβ_1−42_K_3_) was slightly improved to 7.8%, accompanied by a slight increase in purity to 90.2%. This improvement might in part be attributed to the presence of an additional, positively-charged lysine residue at the C-terminus of the peptide, which resulted in an improved solubility. Further bio-testing was unfortunately not undertaken by the group.

## Paradís-Bas et al. (2014)

**Figure 2:**
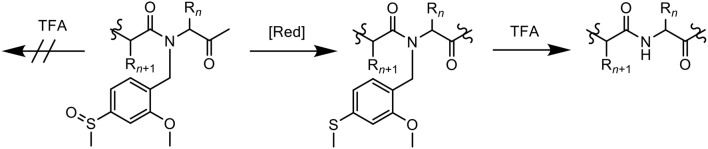
Mmsb, a novel backbone amide-protecting group for the synthesis and purification of “difficult peptide” sequences.

Paradís-Bas et al. introduced 2-methoxy-4-methylsulfinylbenzyl (Mmsb), a novel backbone amide safety-catch protecting group in the synthesis and purification of three “difficult peptide” sequences: H-(Ala)_10_-NH_2_, Ac-(RADA)_4_-NH_2_, and Aβ_1−42_ (Paradís-Bas et al., 2014). Mmsb, which contains an electron-withdrawing sulfoxide that stabilizes a benzyl moiety, is readily reduced to its corresponding electron-donating thioether, which becomes labile to the same benzyl group. With regards to Fmoc/tBu SPPS, Mmsb is stable to TFA, however its reduced form 2-methoxy-4-methylthiobenzyl (Mmtb) is labile to the acid (Figure 2), therefore rendering it a suitable option.

**Scheme 13:**
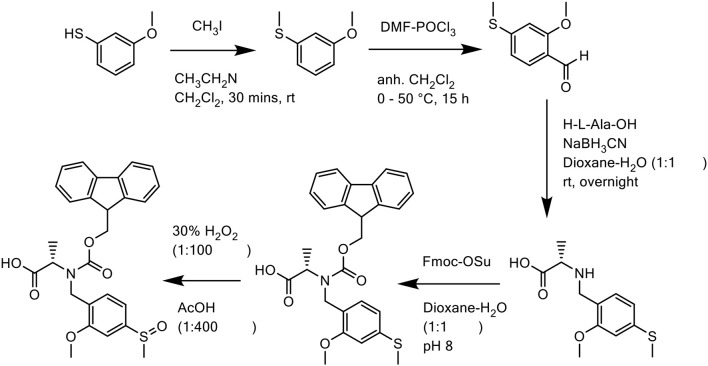
Chemical synthesis of Fmoc-*N*(Mmsb)-Ala-OH.

In all three cases, the Mmsb backbone was introduced into the peptide sequence as its corresponding Fmoc derivative Fmoc-*N*(Mmsb)-Ala-OH. Synthesis of this derivative was undertaken in five steps (Scheme 13): first, commercially available 3-methoxythiophenol was methylated with iodomethane (MeI). Trimethylamine was added dropwise to prevent dialkylation. The alkylated product was subsequently formylated using Vilsmeier reagent, affording 2-methoxy-4-methylthiobenzaldehyde, which was purified by RP-HPLC on an XBridge™ BEH130 C18 column (4.6 × 100 mm, 3.5 μm) in 48% yield. Reductive amination of this aldehyde with the amine of the unprotected alanine residue was achieved in a one-step reaction with NaBH_3_CN in dioxane/H_2_O (1:1 *v/v*). *N*(Mmtb)-Ala-OH was protected by Fmoc using a slight excess of Fmoc-OSu under basic conditions to afford Fmoc-*N*(Mmtb)-Ala-OH, which was again purified by RP-HPLC on an XBridge™ BEH130 C18 column (4.6 × 100 mm, 3.5 μm) to delineate the unreacted alanine. Lastly, oxidation of Fmoc-*N*(Mmtb)-Ala-OH by H_2_O_2_ generated Fmoc-*N*(Mmsb)-Ala-OH in remarkable purity (98.0%). This building block was utilized directly for SPPS of all three peptides.

**Scheme 14:**
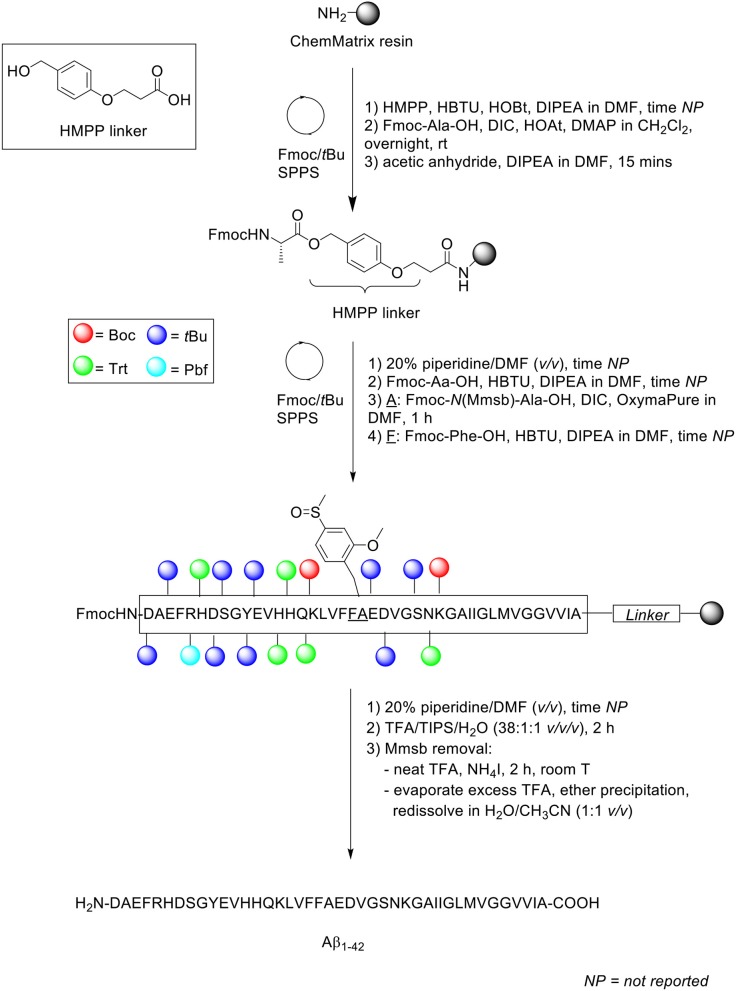
Synthesis of Aβ_1−42_ with Mmsb protecting group by Paradís-Bas et al. (2014).

Peptide synthesis was undertaken on aminomethyl ChemMatrix resin (0.62 mmol/g loading) as solid support at 0.1 mmol scale, which was functionalized with HMPP linker (Scheme 14). Fmoc removal was achieved using 20% piperidine in DMF (*v/v*). Fmoc-AA-OH coupling was performed in the presence of HBTU as coupling reagent, with DIPEA and DMF as solvent. The Fmoc-*N*(Mmsb)-Ala-OH building block, which replaced Ala21 in the Aβ_1−42_ sequence, was coupled using DIC and OxymaPure in DMF for 1 h, followed by sequential washes with DMF and CH_2_Cl_2_, and Kaiser test to confirm completeness of the reaction qualitatively. Fmoc-Phe-OH, the subsequent residue in the sequence, was also coupled manually were coupled manually. The completed peptide chain was cleaved from the resin with TFA/TIPS/H_2_O (38:1:1 *v/v/v*) for 2 h, which demonstrated the stability of Mmsb to acids. Purity of the crude product was relatively low (35%), as determined by RP-HPLC analysis on a Symmetry300™ C4 column (4.6 × 150 mm, 5 μm) at 60°C and flow rate of 1 mL/min. A linear gradient of 10–50% B was used, where solvent B was CH_3_CN + 0.036% TFA, and solvent A was H_2_O + 0.045% TFA. Purification was undertaken by semi-preparative RP-HPLC using a Phenomenex Jupiter C4 column (21 x 150 mm, 10 μm) at a flow rate of 20 mL/min. A linear gradient of 0–10% B over 5 min and 10–50% B over 60 min was used, where solvent B was CH_3_CN + 0.1% TFA and solvent A was H_2_O + 0.1% TFA. Removal of the Mmsb amide protecting group was readily achieved by treatment with neat TFA (1 mg/mL) and ammonium iodine at room temperature for 2 h. Excess TFA was evaporated by gentle N_2_ stream, and the filtrate precipitated in diethyl ether and redissolved in H_2_O/CH_3_CN (1:1 *v/v*), prior to lyophilization.

## Karas et al. (2017)

**Scheme 15:**
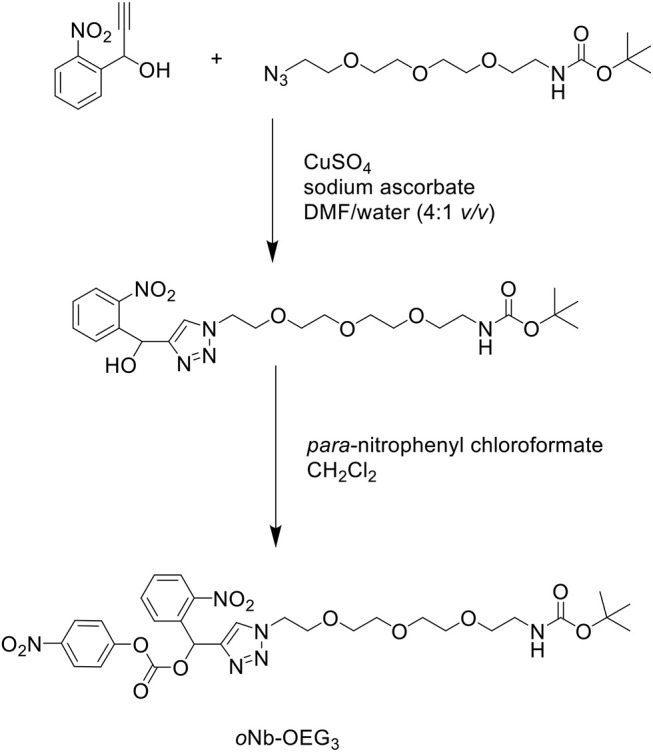
Synthesis of *o*Nb-OEG_3_ solubilizing tag for Aβ_1−42._

In another attempt to improve the solubility properties of Aβ_1−42_, Karas et al. developed a short, monodisperse oligo(ethylene) glycol (OEG)-containing photolabile tag, which was functionalized on residue Lys28 of the Aβ_1−42_ sequence to solubilize the otherwise hydrophobic peptide (Karas et al., 2017). The tag was synthesized in two steps (Scheme 15): an alkyne functional group was incorporated at the benzylic position of an *ortho*-nitrobenzyl (*o*Nb) derivative via a Grignard reaction. This was then reacted with an azide substrate through copper(I)-catalyzed alkyne-azide cycloaddition (CuAAC) in the presence of copper sulfate (CuSO_4_) and sodium ascorbate in DMF/water (4:1 *v/v*) to yield an intermediate, which was activated by *para*-nitrophenyl chloroformate in CH_2_Cl_2_ to afford the desired *o*Nb-OEG_3_ tag.

**Scheme 16:**
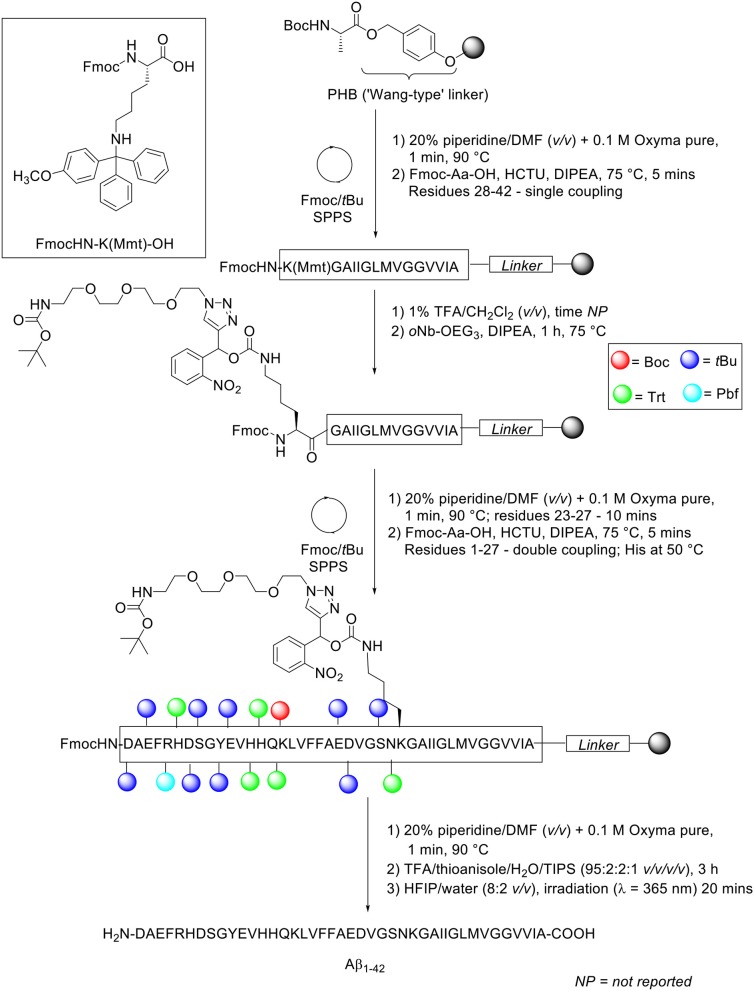
Fmoc/*t*Bu SPPS of Aβ_1−42_ with *o*Nb-OEG_3_ tag functionalized on Lys28 residue by Karas et al. (2017).

The group utilized microwave-assisted Fmoc/*t*Bu SPPS to synthesize their site-specific tagged Aβ_1−42_. Peptide synthesis commenced on TentaGel resin (0.18 mmol/g loading) functionalized with Wang linker, and preloaded with Fmoc-Ala-OH at 0.1 mmol scale (Scheme 16).

The Fmoc protecting group was removed using 20% piperidine in DMF (*v/v*) with 0.1 M Oxyma pure at 90°C for 1 min, except for residues Asp23-Asn27 which were deprotected for 10 min at the same temperature. Fmoc-amino acids (5 eq.) were coupled in the presence of HCTU (5 eq.) and DIPEA (10 eq.). Residues Lys28-Ala42 were coupled once at 75°C, while residues Asp1-Asn27 were double coupled under the same conditions, except for the three sensitive histidine residues, which were double coupled at 50°C. Removal of the monomethoxytrityl (Mmt) side chain protecting group at Lys28 was afforded through sequential treatments with 1% TFA in CH_2_Cl_2_ (*v/v*). The resin bed was then neutralized, and resultant free amine condensed with *o*Nb-OEG_3_ tag (3 eq.) coupled using DIPEA (6 eq.) for 1 h at 75°C to afford the carbamate linkage. The completed peptide chain was cleaved from the resin using TFA/thioanisole/water/TIPS (95:2:2:1 *v/v/v/v*) for 3 h. The TFA filtrate was concentrated *in vacuo*, precipitated in cold ether, and recovered by lyophilization.

A sample of the crude peptide was purified by RP-HPLC using a Phenomenex Kinetex XB-C18 AXIA packed column (100Å, 21.2 × 150 mm, 5 μ) at 60°C. A linear gradient of 20–60% B was employed over 40 min with a flow rate of 5 mL/min. Solvent A was 10 mM ammonium acetate in water (pH 9.2), whereas solvent B was 10 mM ammonium acetate in acetonitrile/water (8:2 *v/v*, pH 9.2). The purified, tagged peptide was afforded at 9.6% yield relative to purified crude weight and >95% purity. The solubilizing tag was subsequently removed by photolysis at 365 nm. The photocleavable-tagged Aβ_1−42_ was first dissolved in HFIP/water (8:2 *v/v*), irradiated for 20 min, and then injected directly into RP-HPLC column for purification, which yielded pure Aβ_1−42_ in 60% yield. Unfortunately, the purity of the final product was not reported by the authors. Biophysical characterization of Aβ_1−42_ synthesized using this method was subsequently undertaken. Comparative TEM images between tagged Aβ_1−42_ and native Aβ_1−42_ obtained following photolysis showed a suppression in fibril formation, which was further quantified by a lower ThT fluorescence, after incubation in phosphate buffered saline (PBS) for 48 h at 37°C. Thus, incorporation of this tag into the Aβ_1−42_ peptide sequence was successfully shown by this group to improve its solubility properties, affording the desired product at higher yield and purity.

## Kasim et al. (2019)

**Scheme 17:**
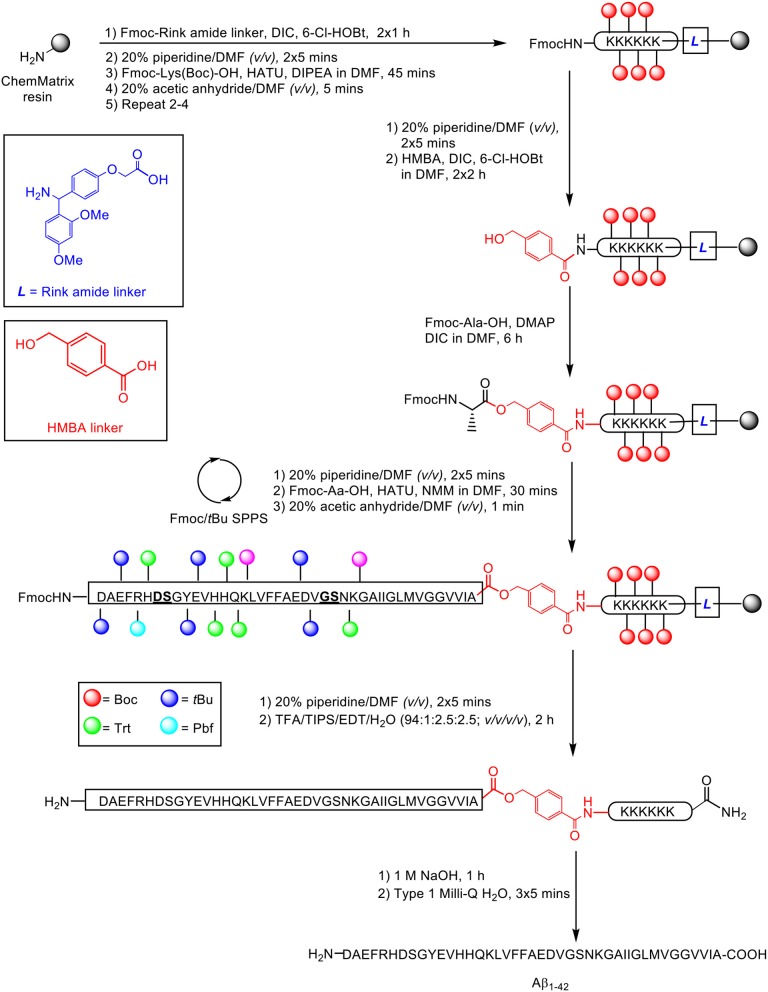
Fmoc/*t*Bu SPPS of Aβ_1−42_ on a double linker system, employing pseudoproline dipeptides as aggregation disruptors by Kasim et al. (2019). Residues in bold and underlined indicate site of pseudoproline incorporation.

Recently, our group published an improved methodology for the synthesis and efficient characterization of the Aβ_1−42_ peptide (Scheme 17; Kasim et al., 2019).

The proposed synthetic strategy utilized a double linker system, which has previously enabled efficient synthesis of the aggregating cancer protein NY-ESO1 (Harris and Brimble, 2009) and peptide hormone vesiculin (Williams et al., 2013). Peptide synthesis commenced on the low-loading ChemMatrix resin (0.64 mmol/g loading) at 0.1 mmol scale, using Fmoc/*t*Bu SPPS. Fmoc-Rink amide linker (4 eq.) was first anchored on resin in the presence of DIC (4 eq.) and 6-chlorohydroxybenzotriazole (6-Cl-HOBt, 4 eq.) as coupling reagents, followed by sequential assembly of the hexalysine tag using HATU (4.6 eq.) and DIPEA (10 eq.), and coupling of the bifunctional 4-hydroxymethylbenzoic acid (HMBA) linker (4 eq.) using DIC (4 eq.) and 6-Cl-HOBt (4 eq.), completing the double linker construct. The first C-terminal residue alanine was then esterified on HMBA linker using Fmoc-Ala-OH (2 eq.), DIC (2 eq.) and DMAP (0.9 eq.). Peptide chain elongation was achieved using Fmoc-Aa-OH (5 eq.), HATU (4.6 eq.), and NMM (10 eq.), followed by cleavage off resin using TFA/TIPS/EDT/water (94:1:2.5:2.5 *v/v/v/v*). The TFA filtrate was evaporated using a gentle nitrogen stream, precipitated in cold ether, and recovered by lyophilization (56% yield based on 0.1 mmol ChemMatrix loading).

Purification of the crude product was undertaken by RP-HPLC using a semi-preparative Zorbax 300SB-C3 column (9.4 × 250 mm, 5 μm) under conventional conditions (acidic mobile phases, room temperature). A linear gradient of 1–61% B was employed over 60 min, where solvent B was 0.1% TFA in acetonitrile and solvent A was 0.1% TFA in water. One highlight of this methodology is that it was possible for purification of the crude product to be carried out on a relatively large scale, up to 90 mg in a single batch. The presence of the lysine tag is proposed to facilitate this process. The purified peptide-linker was subsequently treated with 1 M sodium hydroxide for 1 h to hydrolyse the base-labile ester bond between alanine and HMBA linker. The liberated linker was then readily dissolved following sequential washes with ultrapure water, recovering the peptide quantitatively without the need for any additional HPLC purification steps, and affording the desired peptide at 92% purity. Aβ_1−42_ was then subjected to biophysical assessment by TEM imaging, ThT assay, and circular dichroism (CD) spectroscopy. The resulting TEM images indicated that the peptide did form fibrils over time, and possessed a concentration-dependent aggregation profile in response to ThT binding, as well as a secondary structure composition of predominantly beta-sheets and random coils, as quantified by CD. Therefore, Aβ_1−42_ synthesized through this methodology is sufficiently bioequivalent for use in further *in vitro* and *in vivo* biological studies.

## Summary

In this review, a selection of methodologies designed to facilitate the efficient synthesis of Aβ_1−42_ peptide has been presented and discussed in great depth. Essential details about each protocol are summarized within Table 2, providing a convenient point of reference for comparative purposes. In terms of SPPS methodology, the majority opted for Fmoc/*t*Bu SPPS. While there is a relatively extensive range of resins and linkers employed, these in general possess a low degree of substitution, which limited steric interference as the desired peptide elongated. With regards to purification of the crude material, there is also a great deal of variety in the chromatographic column used, but not so much with the solvent system, primarily 0.1% TFA in acetonitrile and 0.1% TFA in water, and temperature; most groups reviewed herein carried out their purification at room temperature. Yields of the final product are expectedly low, but collectively high in purity. Lastly, although this was not generally undertaken, groups that performed bioequivalence testing on their synthesized Aβ_1−42_ all reported on a high similarity with the endogenous peptide, in terms of its biological profile. With regards to this, we propose that it should be made necessary for any future work in this field to include evidence of bioequivalence.

**Table 2:** Comparative table of methods and conditions employed for the synthesis and purification of Aβ_1−42_ peptide.

**References**	**Method**	**Resin**	**Linker**	**Purification**			**Purified yield, purity (%)**
				**Column**	**Solvents**	**Temp. (^**°**^C)**	
Burdick et al., 1992	Fmoc/*t*Bu	PEG-PS	*p*-alkoxybenzyl	C4	CH_3_CN (0.1% TFA)/H_2_O (0.1% TFA)	RT	*NP, NP*
Hendrix et al., 1992	Boc/Bzl	Merrifield	Kaiser Oxime	*NP*	*NP*	RT	*NP*, >90
Milton et al., 1997	Fmoc/*t*Bu	PEG-PS	*p*-alkoxybenzyl	C4	CH_3_CN (0.1% TFA)/H_2_O (0.1% TFA)	RT	28, *NP*
Fukuda et al., 1999	Fmoc/*t*Bu	PEG-PS	*p*-alkoxybenzyl	C18	CH_3_CN (0.1% NH_4_OH)/H_2_O (0.1% NH_4_OH)	RT	10, *NP*
Tickler et al., 2001	Fmoc/*t*Bu	PEG-PS	HMPA	C4	CH_3_CN (0.1% TFA)/H_2_O (0.1% TFA)	60	17, *NP*
Carpino et al., 2004	Fmoc/*t*Bu	TentaGel	HMPB	*NP*	*NP*	RT	*NP, NP*
Kim et al., 2004	Boc/Bzl	Aminomethyl PS	PAM	diphenyl	CH_3_CN (0.09% TFA)/H_2_O (0.09% TFA)	RT	*NP, NP*
Sohma et al., 2005	Fmoc/*t*Bu	Aminomethyl PS	2-CTC	C18	CH_3_CN (0.1% TFA)/H_2_O (0.1% TFA)	40	*NP*, >95
García-Martín et al., 2006	Fmoc/*t*Bu	ChemMatrix	HMPP	C8	CH_3_CN (0.1% TFA)/H_2_O (0.1% TFA)	60	*NP, NP*
Zarándi et al., 2007	Fmoc/*t*Bu	PS	Wang	C4	CH_3_CN/H_2_O (0.1% TFA)/H_2_O (0.1% TFA)	RT	*NP*, >95
Bacsa et al., 2010	Fmoc/*t*Bu	ChemMatrix	Rink amide	C4	CH_3_CN (0.1% TFA)/H_2_O (0.1% TFA)	60	*NP, NP*
Collins et al., 2014	Fmoc/*t*Bu	PEG-PS	PAL	C18	CH_3_CN (0.1% CH_2_O_2_)/H_2_O (0.1% CH_2_O_2_)	RT	*NP, NP*
Chemuru et al., 2014	Fmoc/*t*Bu	PEG-PS	*NP*	C3	CH_3_CN (0.05% TFA)/H_2_O (0.05% TFA)	RT	7.8, 90.2
Paradís-Bas et al., 2014	Fmoc/*t*Bu	ChemMatrix	HMPP	C4	CH_3_CN (0.1% TFA)/H_2_O (0.1% TFA)	RT	*NP*, 90
Karas et al., 2017	Fmoc/*t*Bu	TentaGel	PHB	C18	CH_3_CN/H_2_O (10 mM NH_4_OAc)/H_2_O (10 mM NH_4_OAc)	60	*NP, NP*
Kasim et al., 2019	Fmoc/*t*Bu	ChemMatrix	Rink amide, HMBA	C3	CH_3_CN (0.1% TFA)/H_2_O (0.1% TFA)	RT	8.6, 92

*NP, not reported*.

## Conclusions and Future Directions

At the present time, it is clear that significant strides have been taken toward the efficient synthesis and characterization of the amyloidogenic Aβ_1−42_ peptide since its maiden synthesis by Burdick et al. nearly three decades ago. Advancements in the field of “difficult peptide” synthesis, more specifically Aβ_1−42_, translates to its production in ample quantities and, perhaps most importantly, with a purity that mirrors Aβ_1−42_ isolated from natural sources, such as post-mortem human brain tissue. Identification of the key challenge associated with its preparation, namely the propensity to aggregate both on resin during SPPS and in solution, has propelled the developed of methods which have effectively addressed this issue. The so-called “Aβ_1−42_ problem” can thus be mitigated indirectly through optimization of the synthetic protocol employed, such as the use of PEG-based ChemMatrix resin (García-Martín et al., 2006; Kasim et al., 2019) to minimize the degree of steric interference as peptide elongation progresses, or through careful, considered choice of solvents and reagents that facilitate a more complete deprotection and coupling of amino acid residues. Furthermore, direct chemical modifications on the peptide sequence, achieved for instance through the introduction of removable solubilizing tags (Chemuru et al., 2014; Karas et al., 2017; Kasim et al., 2019) has also been proven to be an equally effective means. Thus, it would be a most logical deduction to propose that a combination of both approaches should produce a synergistic effect.

The extensive variety of methods that have been employed thus far, however, raises the curious question as to whether there is a need for a streamlined, “one-size-fits-all” protocol for the efficient synthesis of Aβ_1−42_. We argue that such a generalized method is not required. Rather, any method designed specifically for the preparation of this amyloidogenic peptide must be relatively easy to reproduce by any standard peptide synthesis laboratory. With respect to this, the reagents employed should incur a reasonable cost and be easily accessible. Consequently, Aβ_1−42_ synthesis using Boc/Bzl SPPS might not be especially favorable given the necessity for specialized equipment, some of which have their use restricted by local authorities. Of course, there is also the general safety issue pertaining to the use of strong acids such as HF as an essential component of this strategy. Perhaps, then, the future of Aβ_1−42_ lies on the more routinely employed Fmoc/*t*Bu SPPS. Ultimately, any method employed should enable preparation of multi-milligram amounts of this peptide, and at a purity level that is identical to the endogenous peptide.

While most of the studies reviewed herein did not report their final Aβ_1−42_ purity, satisfactory evidence was provided in general as a proxy, in most cases through comparative assessment with commercially available Aβ_1−42_, as well as *in vitro* studies employing the synthesized Aβ_1−42_. The latter is perhaps of greater importance than the former, as they provide proof of bioequivalence, and suggests that even though the synthesized peptide may not be of especially high purity, it is still capable of behaving in a manner identical to endogenous Aβ_1−42_, which we believe should be considered as a hallmark of a successful synthesis.

## Author Contributions

JK and IK conceived the review article. PH and MB proofread and provided feedback on overall structure and content of the manuscript.

### Conflict of Interest Statement

The authors declare that the research was conducted in the absence of any commercial or financial relationships that could be construed as a potential conflict of interest.
